# No association between genetically predicted C-reactive protein levels and colorectal cancer survival in Korean: two-sample Mendelian randomization analysis

**DOI:** 10.4178/epih.e2023039

**Published:** 2023-03-22

**Authors:** Chang Kyun Choi, Jung-Ho Yang, Min-Ho Shin, Sang-Hee Cho, Sun-Seog Kweon

**Affiliations:** 1 Department of Preventive Medicine, Chonnam National University Medical School, Hwasun, Korea; 2 Department of Hematology-Oncology, Chonnam National University Hwasun Hospital, Hwasun, Korea

**Keywords:** Colorectal neoplasm, C-reactive protein, Survival analysis, Mendelian randomization analysis

## Abstract

**OBJECTIVES:**

Elevated C-reactive protein (CRP) levels are associated with an increased risk for colorectal cancer (CRC), as well as a poor prognosis, but it remains unclear whether these associations are causal. This study examined the potential causality between CRP levels and CRC survival using 2-sample Mendelian randomization (MR).

**METHODS:**

From the Korean Genome and Epidemiology Study, a genome-wide association study (n=59,605), 7 single-nucleotide polymorphisms (SNPs) related to log_2_-transformed CRP levels were extracted as instrumental variables for CRP levels. The associations between the genetically predicted CRP and CRC-specific and overall mortality among CRC patients (n=6,460) were evaluated by Aalen’s additive hazard model. The sensitivity analysis excluded a SNP related to the blood lipid profile.

**RESULTS:**

During a median of 8.5 years of follow-up, among 6,460 CRC patients, 2,676 (41.4%) CRC patients died from all causes and 1,622 (25.1%) died from CRC. Genetically predicted CRP levels were not significantly associated with overall or CRC-specific mortality in CRC patients. The hazard difference per 1,000 person-years for overall and CRC-specific mortality per 2-fold increase in CRP levels was -2.92 (95% confidence interval [CI], -14.05 to 8.21) and -0.76 (95% CI, -9.61 to 8.08), respectively. These associations were consistent in a subgroup analysis according to metastasis and a sensitivity analysis excluding possible pleiotropic SNPs.

**CONCLUSIONS:**

Our findings do not support a causal role for genetically predisposed CRP levels in CRC survival.

## GRAPHICAL ABSTRACT

[Fig f1-epih-45-e2023039]

## INTRODUCTION

C-reactive protein (CRP) is widely used as a clinical parameter to measure inflammatory status, and numerous observational studies have reported associations between elevated CRP levels and the risk of colorectal cancer (CRC) incidence [1,2], population mortality due to CRC [3,4], and the clinical prognosis of CRC patients, including death [5,6], metastasis [5,7], postoperative complications [8,9], and recurrence [5,10,11]. Three systematic reviews and meta-analyses have provided up-to-date information on the association between elevated CRP levels and a poor prognosis of CRC [10,12,13]. For various other solid tumors, as well as CRC, CRP is a variable that must be included when constructing a prognostic prediction model [10], including the Glasgow Prognostic Score, which is known to effectively predict the prognosis of CRC patients [14]. However, in these observational studies, it is difficult to differentiate between the deterioration of cancer-related inflammation and the clinical impact of elevated CRP levels themselves, because most studies analyzed peri-treatment CRP levels. The best timing for evaluating CRP-related markers remains unclear [6]. In addition, since the association between cancer progression and an immune response is often bidirectional and multifactorial, it is difficult to avoid reverse causality and residual confounding effects in observational studies [15]. To overcome these limitations and clarify the true causality, Mendelian randomization (MR) using genotypes as instrumental variables has been widely applied. Because genetic variants are randomly allocated by Mendel’s law, an MR study using genetic variants as instrumental variables can be independent of potential confounders and can exclude the possibility of reverse causality. However, constructing large data sets with intermediate phenotypes and genetic instruments is challenging due to the high cost of measurements and/or the lack of suitable biological specimens. In this context, 2-sample MR can evaluate the association between exposure and outcome using 2 independent existing genome-wide association studies (GWASs), and 2-sample MR is steadily becoming more common in research using MR analysis [16].

GWASs have reported that several single-nucleotide polymorphisms (SNPs) were associated with CRP levels [17-19]; in the largest recent GWAS, those SNPs explained about 7.0% of the variance in CRP levels [17]. However, limited epidemiological data have been reported on the association between CRP-related genetic variants and the prognosis of CRC, and the findings are still inconsistent [20-23].

The International Survival Analysis in Colorectal Cancer Consortium recently reported that genetically predicted CRP levels were not significantly associated with CRC-specific mortality in a GWAS of 16,918 European CRC cases [24]. However, since CRP levels vary among ethnicities [25] and several SNPs were found to be related to CRP levels only in a GWAS of East Asians [19,26,27], there remains a need for further research to clarify the association between CRP levels and survival of CRC in other ancestries. Therefore, we evaluated the causal role of genetically predicted CRP levels in the survival of CRC in Koreans by conducting a 2-sample MR study with representative Korean GWAS datasets.

## MATERIALS AND METHODS

### Sources of the C-reactive protein genome-wide association study data

Supplementary Material 1 shows the flow chart of the CRP GWAS. The association between SNPs and CRP levels was determined using the GWAS dataset from the Korean Genome and Epidemiology Study (KoGES) [28], a consortium project consisting of 6 prospective cohort studies supported by government funding. Over 223,000 participants have been recruited, with 72,298 from population-based studies (58,700 from the KoGES Health Examinee [KoGES_HEXA] study, 8,105 from the KoGES Cardiovascular Association Study [KoGES_CAVAS], and 5,493 from the KoGES Ansan and Ansung Study) who provided epidemiological information and genome-wide arrays after a quality control procedure. The KoGES_CAVAS and KoGES Ansan and Ansung Study consisted of community inhabitants, while the KoGES_HEXA study included participants recruited from the national health examinee registry. From the original sample, 10,358 participants whose serum CRP level was not measured, 146 participants with a serum CRP level ≥ 10 mg/L, 2,462 participants with a previous history of cancer, and 81 participants with missing values for a previous history of cancer were excluded from the analysis. Consequently, 59,605 participants were included in the final analysis (Supplementary Material 2). Genomic DNA was extracted from peripheral blood, and the GWAS was conducted using the Korean Biobank Array (K-CHIP) customized for the Korean population. Details on genotyping, GWAS quality control, and imputation have been described elsewhere [29]. K-CHIP contains 833,535 SNPs, including 89,413 SNPs present in East Asians. Imputation was conducted using the 1000 Genomes Phase 3 dataset of the East Asian population as a reference panel.

### Source of the colorectal cancer genome-wide association study data

The CRC GWAS data were obtained from the Hwasun Cancer Epidemiology Study-Colon and Rectum Cancer (HCES-CRC). The Hwasun Cancer Epidemiology Study (HCES) is a hospital-based case-control study aiming to identify serologic and genetic risk factors for multiple cancers, including esophageal [30], breast [31], gastric [32], and colorectal cancers [33]. The HCES-CRC consisted of 7,089 hospital-based CRC cases and 4,979 populationbased cancer-free controls. Details of genotyping and GWAS quality control have been described elsewhere [33]. The baseline characteristics of the CRC GWAS are presented in Supplementary Material 3. In brief, the subjects were patients diagnosed with histologically confirmed CRC at Chonnam National University Hwasun Hospital between 2004 and 2014. Germline DNA genotyping was performed using the Infinium OncoArray-500K BeadChip (Illumina Inc., San Diego, CA, USA) in 3,158 CRC cases, and the Infinium Multi-Ethnic Global BeadChip (MEGA, Illumina Inc.) in 3,465 cases. Of those, 163 cases without information on the tumor, node, metastasis stage were excluded, and 6,460 CRC cases were finally included in the analysis. The cause and date of death were obtained from the National Statistical Office. The date of death was ascertained until December 31, 2020. The cause of death was coded according to the International Classification of Diseases, 10th revision. The details of the HCES-CRC and imputation procedure have been described previously [34]. The analysis included SNPs with an info score greater than 0.4.

### Associations between genetic variants, C-reactive protein levels, and colorectal cancer survival

This study consisted of a discovery cohort of 47,258 individuals from KoGES_HEXA and a replication cohort of 12,347 individuals from KoGES_CAVAS and the KoGES Ansan and Ansung Study. Multivariate linear regression was performed to evaluate the association between genetic variants and log_2_-transformed serum CRP levels. Age, sex, survey year, and the assessment centers of cohort studies were adjusted. The first 10 principal components were also adjusted to correct for the possible population structure in the GWAS. The statistical analyses were performed using PLINK version 1.90b6.0 (https://www.cog-genomics.org/plink/). SNPs with a minor allele frequency (MAF) < 0.05 were excluded from the analysis, leaving 1,859 SNPs significantly associated with log_2_-transformed serum CRP levels in KoGES (p< 5× 10^-8^). We used a linkage disequilibrium (LD)-based clumping cut-off of r^2^ <0.001 and a window size of 10,000 kb directly from the KoGES genotyping data. The results of the discovery and replication phases are presented in Supplementary Material 4. Discovery analysis identified 13 significant SNPs associated with serum CRP levels, of which 7 were replicated. The replicated SNPs were rs2794520 near *CRP*, rs12133641 in *IL6R*, rs71086917 in *LINC02819*, rs1260326 in *GCKR*, rs7383869 near *IL6*, rs79320731 in *HNF1A*, and rs429358 in *APOE*.

The association between selected SNPs and log_2_-transformed serum CRP levels was re-evaluated in a pooled analysis, and these results were used for the MR study. The minimum value of the F-statistics of the selected SNPs was 61.2, and it was expected that the bias by weak instruments in the main analysis would not be significant.

Because Aalen’s additive hazard model preserves linearity, it can be used in 2-sample MR analysis regardless of the proportional hazard assumption [35]. Therefore, we conducted Aalen additive hazard regression to evaluate the association between CRP-related SNPs and CRC survival using the R package “timereg.” Statistical analyses were performed using R version 4.2.0 (R Foundation for Statistical Computing, Vienna, Austria).

### Two-sample Mendelian randomization and genetic risk score

We estimated genetically predicted CRP levels and CRC survival using the inverse-variance weighted (IVW) method using the R package “MendelianRandomization” [36].

The estimated associations of genetically predicted CRP levels with hazard differences (HDs) in mortality were expressed with respect to a 2-fold increase in the serum CRP level.

Seven SNPs were selected as instrumental variables to calculate the weighted genetic risk score (GRS) for the log_2_-transformed serum CRP level. Supplementary Material 5 shows the distribution of the weighted GRS for CRP and CRC GWAS. In PLINK, the weights are the estimated beta coefficients associated with each copy of the minor allele in a linear regression analysis. The mean GRS per non-missing genetic marker was calculated and the GRS was divided into quintiles (Q1 to Q5).

### Statistical power

To the best of our knowledge, there is no available tool to estimate statistical power for survival outcomes in MR. Instead, we used a conservative tool that considered binary survival outcomes [37]. Of a total of 6,460 CRC cases, 2,676 (41.4%) deaths occurred over an 8-year follow-up period. In previous meta-analysis of CRC patients, the hazard ratios (HRs) for overall survival of elevated CRP levels and CRP-to-albumin ratio were 2.04 [12] and 2.03 [13], respectively. We had more than 90% power to detect an odds ratio (OR) of 1.50 for the association between CRP levels and overall mortality at a significance level of 0.05, assuming that the GRS would explain 4.0% of the variance in CRP levels.

In addition, we ran a simulation using an additive hazards model for power calculation. With 6,460 CRC cases and 2,676 deaths accrued over an 8-year follow-up, the population-averaged hazard was estimated to be 2,676/(6,460× 8)= 0.052 per person-year (PY). We had at least 90% power to detect a 50.0% difference in hazard (HD, 0.026) for every 1 standard deviation (SD) increase in log_2_-transformed CRP levels, assuming that 4.0% of the variance of CRP was explained by the GRS. The R code for the simulation was modified from the R code in the study of Hua et al. [24].

### Sensitivity analysis

MR relies on 3 assumptions. First, genetic variants are associated with the exposure (CRP). Second, genetic variants are not associated with potential confounders. Third, genetic variants are not directly associated with the outcome (death), except through the exposure (a lack of horizontal pleiotropic effects). In our study, the selected SNPs were validated in the CRP GWAS and were independent of each other (not in LD). In addition, using Phenoscanner, a database of GWAS results, the pleiotropic effects of the selected SNPs were checked [38], and an association of rs429358 near *APOE* with the blood lipid profile was reported [39,40]. Therefore, we performed an additional sensitivity analysis excluding rs429358.

For 2-sample MR, 3 sensitivity analyses (simple and weighted median, and MR-Egger regression) were performed. Although the IVW method is sensitive to violations of the assumption regarding pleiotropy, the results from the simple and weighted median are consistent even when up to 50% of the information comes from invalid instrumental variables [41]. The intercept estimated from the MR-Egger regression provides an estimate of the horizontal pleiotropic effect [42].

### Ethics statement

The KoGES was reviewed and approved by the Korea Centers for Disease Control and Prevention in Korea (IRB No. 2015-08EXP01-C-A and 2016-02-20-C-A). Informed consent was obtained from the participants.

The HCES-CRC was reviewed and approved by the Chonnam National University Hwasun Hospital Institutional Review Board (IRB No. CNUHH-2020-063). All patients and controls gave informed consent to study participation at the time of peripheral blood collection.

## RESULTS

As shown in Table 1, SNPs in 7 loci were associated with log_2_-transformed CRP levels, as follows: rs2794520 near *CRP* (p=7.23×10^-200^), rs12133641 in IL6R (p=3.52×10^-47^), rs71086917 in *LINC02819* (p=6.54×10^-16^), rs1260326 in GCKR (p=1.31×10^-29^), rs7383869 near IL6 (p=3.53×10^-37^), rs79320731 in HNF1A (p=1.22× 10^-99^), and rs429358 in APOE (p=3.53×10^-125^). The estimated log_2_-transformed CRP variance explained by the selected SNPs was 4.0%.

Among these SNPs, rs2794520 (*CRP*) [17], rs12133641 (*IL6R*) [27,43], rs1260326 (*GCKR*) [39], and rs429358 (*APOE*) [17] have been reported to be associated with CRP levels in previous studies. For rs7383869 (*IL6*) and rs79320731 (*HNF1A*), the LD blocks of those SNPs contained rs2097677 (R^2^ = 0.42) and rs7310409 (R^2^ = 0.90), respectively, as reported by a previous GWAS in the Japanese population [26].

There are 2 possible explanations for the association between the remaining novel SNP, rs71086917 (*LINC02819*), and CRP levels. First, although, to our knowledge, an association between *LINC02819* and CRP has not been reported in previous studies, rs10908724 in *LINC02819* and in the LD block for rs71086917 (R^2^ = 0.180) was related to MCP-1 [44], which mediates the chemotaxis of CRP [45]. However, since the results of the GWAS do not reveal the function of the SNP, the association between the regulation of *LINC02819* and CRP or MCP-1 still needs to be evaluated. Second, rs10908724 may be a proxy SNP for rs3093068 (CRP) identified in a previous GWAS [43]. In our genotype data, rs10908724 was correlated to rs3093068 (R^2^ = 0.02).

Table 2 presents the effects of genetically predicted CRP levels on the risk of death using summary statistics and the GRS. The effects estimated by the IVW method showed that a 2-fold increase in serum CRP levels was not significantly associated with the risk of overall or CRC-specific mortality (HD per 1,000 PY: -2.92 and -0.76, respectively; 95% CI, -14.05 to 8.21 and -9.61 to 8.08, respectively). Furthermore, the results of sensitivity analyses using the simple median and median weighted estimation were consistent with the main results. The MR-Egger intercept showed no significant evidence of pleiotropic effects (p= 0.669 for overall mortality and p= 0.876 for CRC-specific mortality). These non-significant results were similar in the sensitivity analysis that excluded the SNP related to the blood lipid profile. The scatter plot of SNP-specific associations with CRC-specific survival against coefficients of SNP-CRP associations and the regression line depicting the association between genetically predicted CRP levels and survival are visualized in Supplementary Materials 6 and 7, respectively. The linear association between a 1-SD increment of the GRS for CRP levels was not significantly associated with CRC-specific mortality (HD per 1,000 PY, -2.09; 95% CI, -4.26 to 0.08). The GRS for CRP levels demonstrated a significant association with overall mortality (HR per 1,000 PY, -2.09; 95% CI, -5.77 to -0.59). Additionally, compared to the third quintile of the GRS for CRP levels, the HR for the first quintile of the GRS for CRP levels was 8.53 (95% CI, 1.12 to 15.94). However, these associations were not observed in a sensitivity analysis that excluded rs429358, a variant known to be associated with the blood lipid profile.

Table 3 presents the subgroup analysis according to metastasis. In the analyses using summary statistics and the individual GRS, the association between genetically predicted CRP levels and mortality was not significant, regardless of metastasis.

## DISCUSSION

This study did not find evidence for an association between genetically elevated CRP levels and survival among CRC patients in Korea. Consistent results were found in a sensitivity analysis excluding a possible pleiotropic SNP and a subgroup analysis according to metastasis.

The effect of CRP levels on survival was recently evaluated in CRC cases of European ancestry using 2-sample MR [24]. Similar to our results, Hua et al. [24] reported that genetically predicted CRP levels were not associated with CRC-specific mortality regardless of metastasis. Compared to the instrumental variants used by Hua et al. [24], among the 7 CRP-related SNPs used in our study, 2 SNPs (rs2794520 and rs1260326) were consistent, and 3 correlated SNPs (rs12133641, rs79320731, and rs429358) were included in the same LD blocks. In addition, the effecting alleles showed a consistent directionality in terms of their impact on CRP levels. However, the effective allele frequency (EAF) and effect size of selected SNPs were different, which is presumed to be due to ethnic differences. The EAF and effect size of rs1260326 were 0.450 and 0.008, respectively, in our study, compared to 0.610 and -0.050, respectively, in the study of Hua et al. [24]. In particular, rs1880241, which was included in the study of Hua et al. [24] was excluded from our CRP GWAS because of its low MAF in the East Asian population (< 0.01). Nonetheless, we could not find a causal effect of CRP levels on survival in CRC patients in this East Asian population, similar to findings in the previously studied European population.

Although not MR studies, several previous genetic studies have investigated survival in CRC patients. Two studies evaluated associations between CRP-related SNPs and mortality in CRC patients, but the results were also not significant [21,22]. Two GWASs examined survival in CRC patients. In the Scottish Colorectal Cancer Study [46], no variants reached the p-value threshold for statistical significance. In contrast, Phipps et al. [47] reported that rs209489 was associated with poor survival in patients with distant metastatic CRC. However, since rs209489 was not significantly associated with log_2_-transformed CRP levels in our GWAS, we did not consider this SNP.

Regarding the association between CRP levels and CRC survival, contrary to our findings, previous observational studies have reported that high CRP levels were associated with a poor CRC prognosis. A meta-analysis of 21 observational studies reported that an elevated preoperative CRP level was associated with poor survival with pooled HRs of 2.04 (95% CI, 1.45 to 2.85) for overall survival and 4.37 (95% CI, 2.63 to 7.27) for CRC-specific survival [12]. In a study of CRC patients treated with neoadjuvant therapy and surgery, CRP levels were associated with disease-free survival independently of carcinoembryonic antigen levels or resection margins [5]. Another meta-analysis using the CRP-to-albumin ratio had similar results [13]. However, since the treatment, stage, and CRP cut-off varied in those studies, it is difficult to distinguish between the effects of cancer-related inflammation and circulating CRP. The prognostic role of CRP levels in CRC patients remains a matter of debate in studies of cancer-free general populations. In the general population, the effects of cancer-related inflammation on the association between CRP and CRC prognosis would be reduced, although inconsistent findings have been reported regarding a positive association between elevated pre-diagnostic CRP levels and CRC mortality. In the National Health and Nutrition Examination Survey III, CRP levels were positively associated with CRC-specific mortality in the general population [3], while the Apolipoprotein Mortality Risk Study reported a null association [48], and the Copenhagen City Heart Study [49] reported a possible association between baseline CRP levels and CRC-specific mortality in their cohort.

Chronic inflammation induces cancer invasion, progression, and metastasis, and influences the efficacy of chemotherapy and immunotherapy [50]. CRP, which is synthesized in the liver, is an acute-phase protein that reflects inflammation. However, we did not find an effect of genetically predicted CRP levels on CRC survival as a systemic inflammatory mediator, unlike many previous case-control studies [5,7,15], prospective studies [3,4], and meta-analyses [10,12,13]. However, since the potential causal role of CRP was not confirmed in previous Mendelian studies, the observed effect of CRP elevation on CRC mortality may be due to residual effects or reverse causality.

Our study had several limitations. First, since information on recurrence was not available, disease-free survival was not included in the analysis. Second, the batch effect may not have been excluded in CRP and CRC GWAS. Therefore, to minimize the batch effect, we evaluated the association between genetic variants and serum CRP levels by statistically adjusting for survey years and study sites in the CRP GWAS, and the association between genetic variants and survival by statistically adjusting for genotyping arrays in the CRC GWAS. Third, because there were only 804 metastatic CRC cases in our study, a further evaluation is needed to clarify the effect of CRP levels on survival in patients with metastatic CRC. Fourth, to confirm the null association between CRP levels and mortality in CRC patients, future studies with higher statistical power are needed. In particular, to improve the power of the MR analysis, the CRP variance explained by genetic instruments should be discussed in terms of the biological effects of selected SNPs or CRP levels on cancer mortality to compensate for the lack of functional analysis of SNPs.

In summary, we found that genetically predicted CRP levels were not associated with the overall or CRC-specific survival of CRC patients. Therefore, our results suggest that genetically predisposed circulating CRP levels do not play a causal role in the prognosis of CRC.

## Figures and Tables

**Figure f1-epih-45-e2023039:**
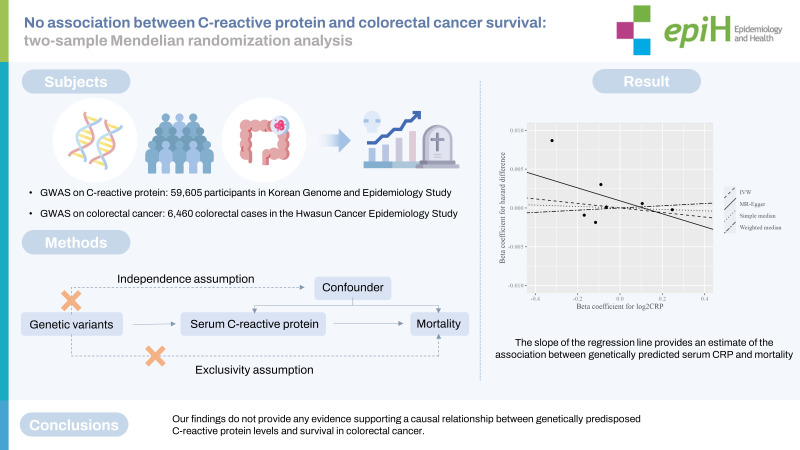


**Table 1.** Selected SNPs used as instrumental variables for log_2_-transformed CRP levels

SNP	Chr	Nearby gene	Position^1^	EA	RA	CRP GWAS^2^				CRC GWAS^3^				
						EAF	F	Beta	p-value	EAF	Overall mortality HD (/1,000 PY)	p-value	CRC mortality HD (/1,000 PY)	p-value
rs2794520	1	*CRP*	159678816	C	T	0.365	961.9	0.247	7.23×10^-200^	0.366	-0.230	0.887	0.018	0.989
rs12133641	1	*IL6R*	154428283	G	A	0.433	197.0	-0.116	3.52×10^-47^	0.429	-1.860	0.210	-0.454	0.701
rs71086917	1	*LINC02819*	159455503	insA	-	0.452	61.2	-0.064	6.54×10^-16^	0.414	0.127	0.938	0.934	0.493
rs1260326	2	*GCKR*	27730940	C	T	0.450	133.0	-0.090	1.31×10^-29^	0.452	3.017	0.050	1.535	0.201
rs7383869	7	*IL6*	22748190	A	G	0.362	164.1	0.105	3.53×10^-37^	0.365	0.571	0.693	0.562	0.620
rs79320731	12	*HNF1A*	121422449	CTGACTGGCACTCAGCA	T	0.455	461.1	-0.169	1.22×10^-99^	0.454	-0.929	0.530	-1.999	0.095
rs429358	19	*APOE*	45411941	C	T	0.096	587.6	-0.321	3.53×10^-125^	0.057	8.684	0.011	7.331	0.014

SNP, single-nucleotide polymorphism; CRP, C-reactive protein; Chr, chromosome; CRC, colorectal cancer; EA, effective allele; EAF, effective allele frequency; GWAS, genome-wide association study; HD, hazard difference; PY, person-year; RA, reference allele.

1 The positions of the SNPs were derived from GRCh37.

2 Age, sex, study centers, survey years, and first 10 principal components were adjusted in all models.

3 Age, sex, genotyping array, tumor, node, metastasis stage, and first 10 principal components were adjusted in all models.

**Table 2.** Mendelian randomization results for the effects of serum CRP levels on overall and CRC-specific mortality

Variables		Main analysis (7 serum CRP level-related SNPs)				Sensitivity analysis (excluding rs429358)			
		Overall mortality	p-value	CRC-specific	p-value	Overall mortality	p-value	CRC-specific mortality	p-value
Using summary statistics^1^									
	IVW	-2.92 (-14.05, 8.21)	0.607	-0.76 (-9.61, 8.08)	0.866	1.11 (-8.18, 10.40)	0.815	2.40 (-4.51, 9.31)	0.497
	Simple median	-0.93 (-13.67, 11.81)	0.886	0.07 (-10.64, 10.78)	0.989	2.25 (-9.74, 14.24)	0.713	2.00 (-7.80, 11.80)	0.689
	Weighted median	1.43 (-8.96, 11.81)	0.788	1.70 (-7.00, 10.41)	0.701	3.39 (-7.00, 13.78)	0.523	2.71 (-5.97, 11.39)	0.540
	MR-Egger intercept	0.92 (-3.29, 5.12)	0.669	0.27 (-3.11, 3.65)	0.876	-0.57 (-4.21, 3.08)	0.760	-1.02 (-3.52, 1.49)	0.426
Using individual GRS^2^									
	GRS per 1 SD increment	-3.18 (-5.77, -0.59)	0.016	-2.09 (-4.26, 0.08)	0.056	0.17 (-1.82, 2.16)	0.870	0.11 (-1.49, 1.70)	0.897
GRS quintile									
	1st	8.53 (1.12, 15.94)	0.024	5.51 (-0.71, 11.73)	0.082	1.94 (-4.47, 8.34)	0.554	2.39 (-2.17, 6.95)	0.349
	2nd	1.86 (-5.16, 8.88)	0.603	1.04 (-4.85, 6.93)	0.730	-2.15 (-8.56, 4.27)	0.512	-1.41 (-5.96, 3.14)	0.598
	3rd	1.00 (reference)		1.00 (reference)		1.00 (reference)		1.00 (reference)	
	4th	-2.64 (-9.13, 3.85)	0.425	-1.54 (-6.68, 3.60)	0.557	-0.52 (-6.74, 5.70)	0.870	-2.06 (-6.53, 2.40)	0.417
	5th	1.14 (-5.17, 7.45)	0.723	0.99 (-4.12, 6.09)	0.705	2.00 (-4.45, 8.45)	0.554	3.16 (-1.45, 7.77)	0.229

Values are presented as hazard difference per 1,000 person-year (95% confidence interval). CRC, colorectal cancer; CRP, C-reactive protein; GRS, genetic risk score; IVW, inverse-variance weighted method; SD, standard deviation; SNP, single-nucleotide polymorphism.

1 Results are expressed per 2-fold increase in serum CRP levels.

2 Age, sex, genotyping array, and tumor, node, metastasis stage were adjusted.

**Table 3.** Subgroup analysis by metastasis of CRC

Variables		Non-metastatic CRC				Metastatic CRC			
		Overall mortality	p-value	CRC-specific mortality	p-value	Overall mortality	p-value	CRC-specific mortality	p-value
Using summary statistics^1^									
	IVW	1.73 (-6.95, 10.41)	0.697	3.37 (-3.41, 10.15)	0.330	-27.69 (-95.81, 40.43)	0.426	-28.19 (-91.79, 35.42)	0.385
	Simple median	3.57 (-6.96, 14.09)	0.506	2.96 (-5.93, 11.85)	0.514	-24.08 (-121.25, 73.10)	0.627	8.90 (-85.99, 103.79)	0.854
	Weighted median	4.73 (-3.69, 13.16)	0.271	5.11 (-1.29, 11.51)	0.117	-42.26 (-125.36, 40.83)	0.319	-30.04 (-108.51, 48.43)	0.453
	MR-Egger intercept	0.04 (-3.37, 3.44)	0.984	-0.68 (-3.24, 1.89)	0.605	12.62 (-10.94, 36.18)	0.294	12.53 (-9.56, 34.62)	0.266
Using individual GRS^2^									
	GRS per 1 SD increment	-1.74 (-3.95, 0.46)	0.121	-0.45 (-2.20, 1.30)	0.613	-18.65 (-42.48, 5.17)	0.125	-22.65 (-45.57, 0.27)	0.053
GRS quintile									
	1st	3.00 (-3.48, 9.49)	0.364	0.37 (-4.51, 5.26)	0.881	61.88 (-5.52, 129.29)	0.072	60.52 (-4.77, 125.81)	0.069
	2nd	-2.96 (-8.76, 2.84)	0.317	-3.08 (-7.39, 1.22)	0.160	45.84 (-14.95, 106.64)	0.139	37.64 (-21.03, 96.31)	0.209
	3rd	1.00 (reference)		1.00 (reference)		1.00 (reference)		1.00 (reference)	
	4th	-0.76 (-6.00, 4.49)	0.778	0.61 (-3.21, 4.43)	0.755	-31.00 (-80.02, 18.02)	0.215	-30.44 (-72.34,11.46)	0.155
	5th	-0.87 (-6.38, 4.65)	0.758	-0.07 (-3.94, 3.80)	0.972	17.10 (-35.25, 69.44)	0.522	2.43 (-43.66, 48.53)	0.918

Values are presented as hazard difference per 1,000 person-year (95% confidence interval). CRC, colorectal cancer; CRP, C-reactive protein; GRS, genetic risk score; IVW, inverse-variance weighted method; SD, standard deviation.

1 Results are expressed per 2-fold increase in serum CRP levels.

2 Age, sex, and genotyping array were adjusted.

## SUPPLEMENTARY MATERIALS

Supplementary Material 1. Flow chart of GWAS for CRP

Supplementary Material 2. Baseline characteristics of GWAS for CRP

Supplementary Material 3. Baseline characteristics of CRC cases according to genotyping array

Supplementary Material 4. . Summary statistics for SNPs significantly associated with serum CRP in discovery and replication set

Supplementary Material 5. Distribution of weighted GRS for CRP and CRC GWAS (A: GWAS of CRP; B: GWAS of CRC), GRS: genetic risk score; CRP: C-reactive protein; CRC: colorectal cancer; GWAS: genome-wide association study

Supplementary Material 6. Scatter plot of SNP-specific associations with overall (A) and CRC-specific (B) mortality against coefficients of SNP-CRP associations. The slope of the regression line provides an estimate of the association between genetically predicted serum CRP and survival.

Supplementary Material 7. Sensitivity analysis excluding rs429358. Scatter plot of SNP-specific associations with overall (A) and CRC-specific (B) mortality against coefficients of SNP-CRP associations. The slope of the regression line provides an estimate of the association between genetically predicted serum CRP and survival excluding.
