# Supplementary material for: No association between genetically predicted C-reactive protein levels and colorectal cancer survival in Korean: two-sample Mendelian randomization analysis
Source: Epidemiol Health. 2023 Mar 22;45:e2023039. doi: 10.4178/epih.e2023039 (PMC10396808; doi:10.4178/epih.e2023039)
Supplement: Supplementary Material 1. — Flow chart of GWAS for CRP [file epih-45-e2023039-Supplementary-1.docx]

Supplementary Material 1. Flow chart of GWAS for CRP
