# Supplementary material for: No association between genetically predicted C-reactive protein levels and colorectal cancer survival in Korean: two-sample Mendelian randomization analysis
Source: Epidemiol Health. 2023 Mar 22;45:e2023039. doi: 10.4178/epih.e2023039 (PMC10396808; doi:10.4178/epih.e2023039)
Supplement: Supplementary Material 2. — Baseline characteristics of GWAS for CRP [file epih-45-e2023039-Supplementary-2.docx]

**Supplementary Material 2. Baseline characteristics of GWAS for CRP**

|  | KoGES_HEXA | KoGES_CAVAS | KoGES_Ansan and Ansung | P-value |
| --- | --- | --- | --- | --- |
|  | (N=47,258) | (N=7,065) | (N=5,415) |  |
| Age (years) | 53.8 ± 8.0 | 58.6 ± 8.9 | 51.6 ± 8.5 | <0.001 |
| Sex |  |  |  | <0.001 |
| Men | 16,374 (34.6) | 2,722 (38.5) | 2,606 (48.1) |  |
| Women | 30,884 (65.4) | 4,343 (61.5) | 2,809 (51.9) |  |
| CRP (mg/dL) | 0.137 ± 0.339 | 0.168 ± 0.396 | 0.220 ± 0.426 | <0.001 |

CRP: C-reactive protein; GWAS: genome-wide association study; KoGES_HEXA: Korean Genome and Epidemiology Study_health examinee; KoGES_CAVAS: Korean Genome and Epidemiology Study_cardiovascular association study
