# Supplementary material for: No association between genetically predicted C-reactive protein levels and colorectal cancer survival in Korean: two-sample Mendelian randomization analysis
Source: Epidemiol Health. 2023 Mar 22;45:e2023039. doi: 10.4178/epih.e2023039 (PMC10396808; doi:10.4178/epih.e2023039)
Supplement: Supplementary Material 3. — Baseline characteristics of CRC cases according to genotyping array [file epih-45-e2023039-Supplementary-3.docx]

**Supplementary Material 3. Baseline characteristics of CRC cases according to genotyping array**

|  | MEGA | Oncoarray | P-value |
| --- | --- | --- | --- |
|  | (N=3,368) | (N=3,092) |  |
| Follow-up duration (years) | 8.3 ± 5.1 | 8.7 ± 4.7 | 0.005 |
| Death | 1,352 (40.1) | 1,324 (42.8) | 0.029 |
| Age (years) | 63.0 ± 11.4 | 62.7 ± 11.2 | 0.362 |
| Sex |  |  | 0.174 |
| Men | 2,116 (62.8) | 1,993 (64.5) |  |
| Women | 1,252 (37.2) | 1,099 (35.5) |  |
| TNM stage |  |  | < 0.001 |
| In-situ | 202 (6.0) | 47 (1.5) |  |
| I | 751 (22.3) | 600 (19.4) |  |
| II | 873 (25.9) | 897 (29.0) |  |
| III | 1,146 (34.0) | 1,140 (36.9) |  |
| IV | 396 (11.8) | 408 (13.2) |  |

All values were presented as ‘N (%)’ or ‘mean ± standard deviation’.

CRC: colorectal cancer; MEGA: Infinium Multi-Ethnic Global BeadChip Array; Oncoarray: Infinium OncoArray-500K BeadChip:
