# Supplementary material for: No association between genetically predicted C-reactive protein levels and colorectal cancer survival in Korean: two-sample Mendelian randomization analysis
Source: Epidemiol Health. 2023 Mar 22;45:e2023039. doi: 10.4178/epih.e2023039 (PMC10396808; doi:10.4178/epih.e2023039)
Supplement: Supplementary Material 4. — . Summary statistics for SNPs significantly associated with serum CRP in discovery and replication set [file epih-45-e2023039-Supplementary-4.docx]

**Supplementary Material 4. Summary statistics for SNPs significantly associated with serum CRP in discovery and replication set**

| SNP | Chr | Nearby  gene | Position^a^ | EA | RA | Discovery set | Replication set | |
| --- | --- | --- | --- | --- | --- | --- | --- | --- |
|  |  |  |  |  |  | P-value | P-value | Replication ^b^ |
| rs2794520 | 1 | CRP | 159678816 | C | T | 5.25x10^-176^ | 4.08x10^-32^ | O |
| rs12133641 | 1 | IL6R | 154428283 | G | A | 9.44x10^-43^ | 1.23x10^-7^ | O |
| rs71086917 | 1 | LINC02819 | 159455503 | insA | - | 2.19x10^-14^ | 2.59x10^-3^ | O |
| rs2209707 | 1 | LEPR | 65930134 | A | G | 3.39x10^-12^ | 4.03x10^-2^ | X |
| rs1260326 | 2 | GCKR | 27730940 | C | T | 1.04x10^-25^ | 4.18 x10^-6^ | O |
| rs9267444 | 6 | MICB | 31483458 | A | G | 2.56x10^-9^ | 5.91x10^-2^ | X |
| rs7383869 | 7 | IL6 | 22748190 | A | G | 1.84x10^-30^ | 1.16x10^-8^ | O |
| rs397846785 | 10 | EXOC6 | 94797202 | delT | - | 2.72x10^-8^ | 3.87x10^-1^ | X |
| rs79320731 | 12 | HNF1A | 121422449 | CTGACTGGCACTCAGCA | T | 4.47x10^-81^ | 2.42x10^-21^ | O |
| rs28608119 | 15 | ONECUT1 | 53096635 | T | C | 1.89x10^-9^ | 7.03x10^-2^ | X |
| rs429358 | 19 | APOE | 45411941 | C | T | 1.81x10^-107^ | 1.61x10^-22^ | O |
| rs1555837686 | 20 | PCIF1 | 44557216 | insAGAGA | - | 1.71 x10^-8^ | 1.50x10^-1^ | X |
| rs4817983 | 21 | LINC02943 | 40464924 | C | G | 5.02x10^-9^ | 2.12x10^-1^ | X |

Age, sex, study centers, survey years, and first 10 principal components were adjusted in all models.

^a^ Position of the SNPs were derived from GRCh37.

^b^ The Benjamin-Hochberg method was applied for multiple testing corrections.

EA: effective allele; EAF: effective allele frequency; RA: reference allele; SNP: single nucleotide polymorphism
