# Supplementary material for: No association between genetically predicted C-reactive protein levels and colorectal cancer survival in Korean: two-sample Mendelian randomization analysis
Source: Epidemiol Health. 2023 Mar 22;45:e2023039. doi: 10.4178/epih.e2023039 (PMC10396808; doi:10.4178/epih.e2023039)
Supplement: Supplementary Material 5. — Distribution of weighted GRS for CRP and CRC GWAS (A: GWAS of CRP; B: GWAS of CRC), GRS: genetic risk score; CRP: C-reactive protein; CRC: colorectal cancer; GWAS: genome-wide association study [file epih-45-e2023039-Supplementary-5.docx]

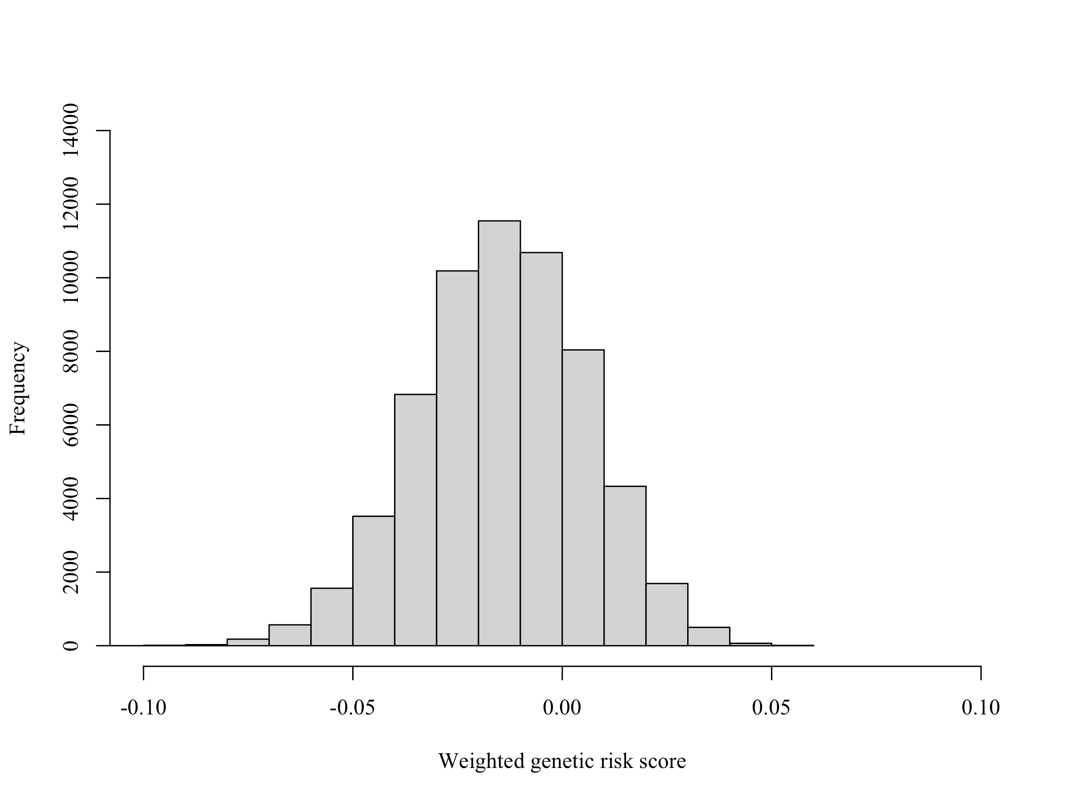

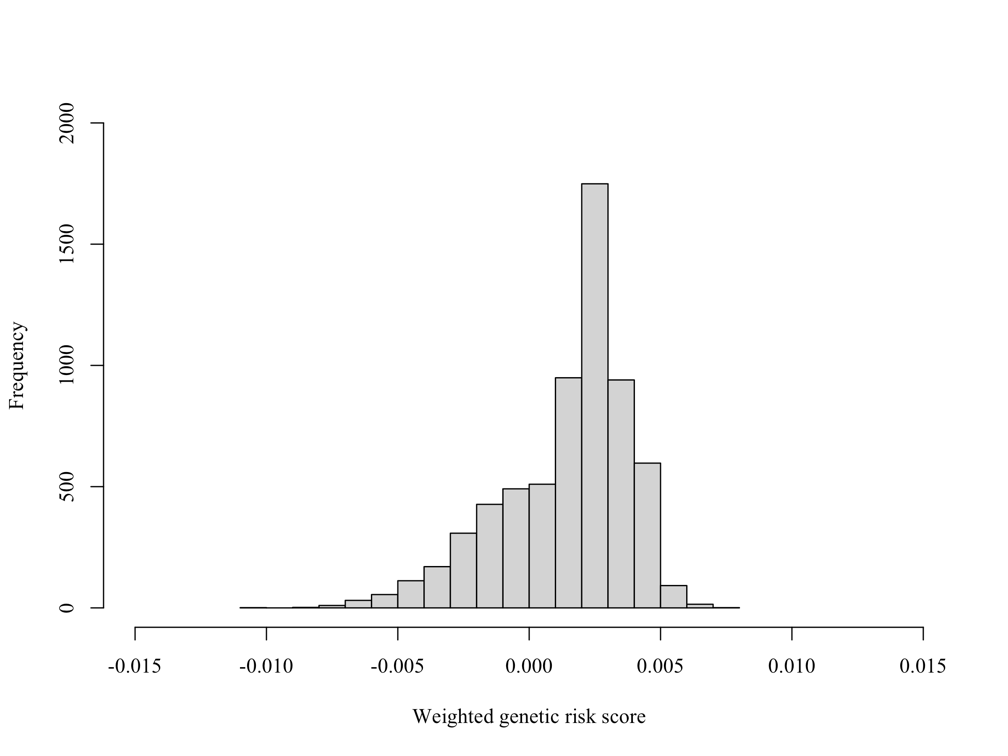


A

B

Supplementary Material 5. Distribution of weighted GRS for CRP and CRC GWAS (A: GWAS of CRP; B: GWAS of CRC), GRS: genetic risk score; CRP: C-reactive protein; CRC: colorectal cancer; GWAS: genome-wide association study
