# Supplementary material for: No association between genetically predicted C-reactive protein levels and colorectal cancer survival in Korean: two-sample Mendelian randomization analysis
Source: Epidemiol Health. 2023 Mar 22;45:e2023039. doi: 10.4178/epih.e2023039 (PMC10396808; doi:10.4178/epih.e2023039)
Supplement: Supplementary Material 7. — Sensitivity analysis excluding rs429358. Scatter plot of SNP-specific associations with overall (A) and CRC-specific (B) mortality against coefficients of SNP-CRP associations. The slope of the regression line provides an estimate of the association between genetically predicted serum CRP and survival excluding. [file epih-45-e2023039-Supplementary-7.docx]

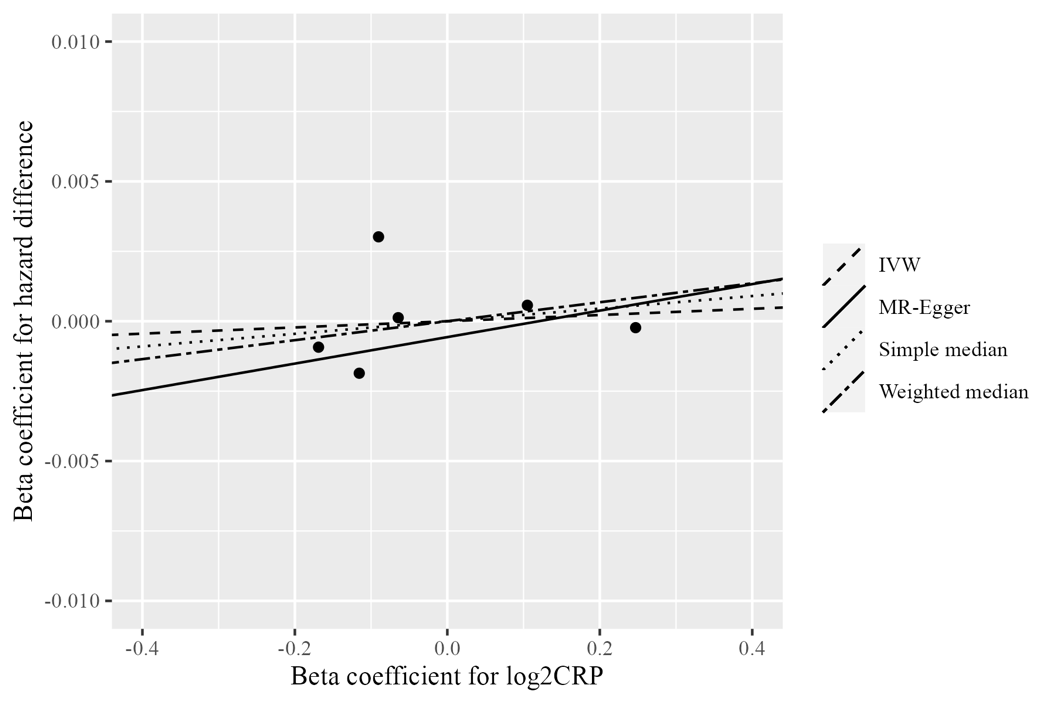

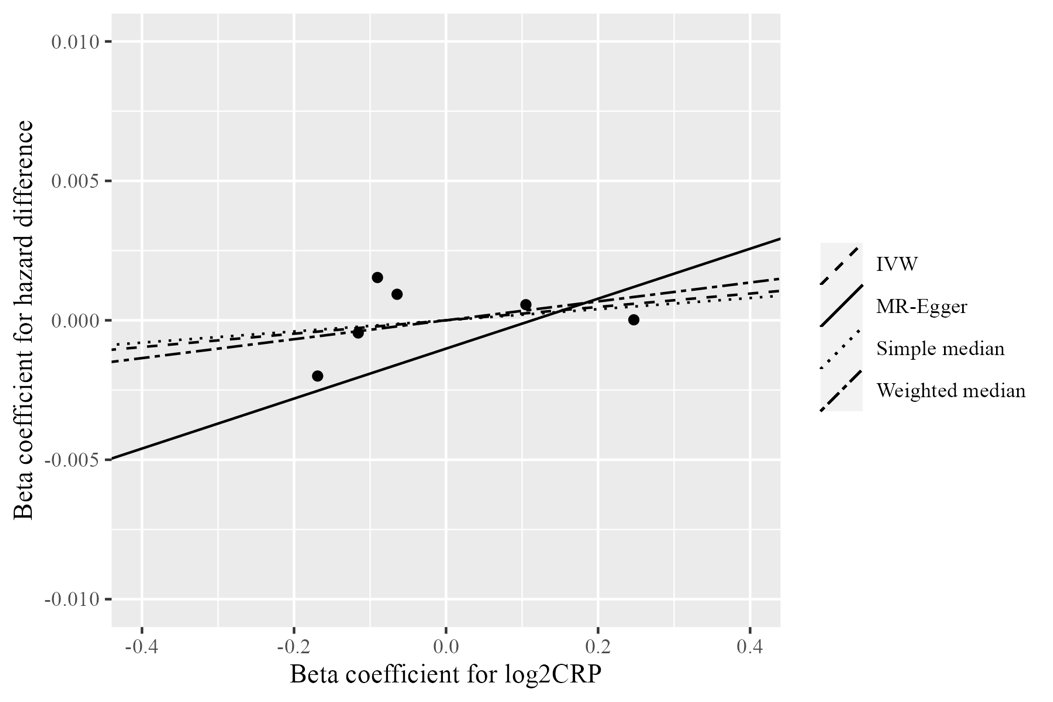


A

B

Supplementary Material 7. Sensitivity analysis excluding rs429358. Scatter plot of SNP-specific associations with overall (A) and CRC-specific (B) mortality against coefficients of SNP-CRP associations. The slope of the regression line provides an estimate of the association between genetically predicted serum CRP and survival excluding.
